# Know Thy Patient, Know Thy Nudge: Unlocking the Potential of Tailored Nudges Based on Patient Characteristics

**DOI:** 10.1016/j.jacadv.2024.101390

**Published:** 2024-11-11

**Authors:** Atsushi Mizuno, Kei Hirai, Fumio Ohtake

**Affiliations:** a Department of Cardiovascular Medicine, St. Luke’s International Hospital, Tokyo, Japan; b Graduate School of Human Science, Osaka University, Suita, Japan; c Center for Infectious Disease Education and Research (CiDER), Osaka University, Suita, Japan; d Graduate School of Economics, Osaka University, Toyonaka, Japan

**Keywords:** behavioral economics, diabetes, influenza vaccination, nudge, NUDGE-FLU

**Figure:**
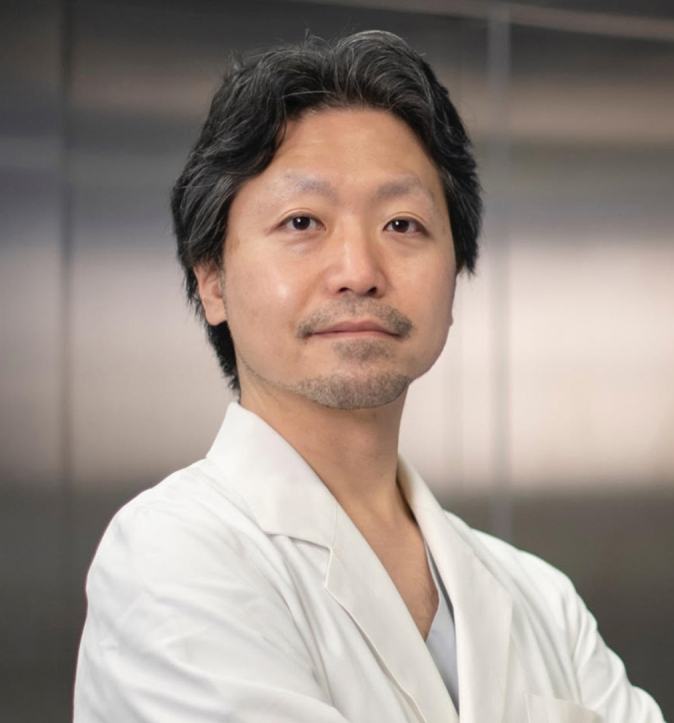

Seasonal influenza infections pose a significant public health challenge, not only because of the increased risk of respiratory infections such as pneumonia but also because of their potential to elevate cardiovascular event risks.^1^ Given these risks, increasing influenza vaccination rates has become a critical public health priority. However, despite these recommendations, vaccination rates remain suboptimal globally, especially among high-risk populations, such as young individuals with heart failure^2^ or diabetes.^3^ This indicates a substantial gap in implementation that must be addressed.

Since Tversky and Kahneman^4^ highlighted the impact of framing on decision-making, extensive research has explored the effects of framing, particularly on health-promoting behaviors. Behavioral economics, which draws on psychological principles, offers a way to address this implementation gap by influencing individual decision-making through strategic interventions. The work of Thaler and Sunstein^5^ highlights the role of behavioral economics, through “nudges”—aspects of choice architecture that alter behavior in predictable ways without forbidding options or significantly changing economic incentives, while preserving their freedom of choice and autonomy. In the context of vaccination, several experimental studies have applied text-based nudges or nudge-type information provision approaches rather than significantly altering the choice architecture through default options, demonstrating their effectiveness.

Milkman et al^6^ pioneered the “megastudy” approach, conducting large-scale randomized controlled trials to systematically test multiple nudge-type interventions based on behavioral economics and psychological theory, identifying effective strategies for influencing behaviors such as increasing influenza vaccination rates in the United States. In Europe, Denmark’s Nationwide Utilization of Danish Government Electronic Letter System for Increasing Influenza Vaccine Uptake (NUDGE-FLU) trial team similarly tested electronic nudge letters using psychological concepts such as reminders and implementation prompts.^7^ Both studies utilized digital technologies such as SMS and smartphone applications and consistently demonstrated the effectiveness of reminder messages. These findings provide a strong foundation for future implementation studies, although further research is needed to understand the influence of different framing messages across various contexts.

In this issue of *JACC: Advances*, Lassen et al^8^ present new findings from a prespecified exploratory analysis of the Nationwide Utilization of Danish Government Electronic Letter System for Increasing Influenza Vaccine Uptake Among Adults With Chronic Disease (NUDGE-FLU-CHRONIC) trial. The trial used a research design that closely resembled that of the NUDGE-FLU trial, the key difference being the target population. While the NUDGE-FLU trial focused on individuals aged 65 years and older, the NUDGE-FLU-CHRONIC trial examined younger and middle-aged adults (age 18-64 years) with chronic diseases (n = 299,881, 19.2% had diabetes) and randomized them into a control group and 6 different message groups to assess influenza vaccination rates. While text-based nudge messaging did not lead to significant changes in outcomes such as cardiovascular and respiratory hospitalizations, all-cause hospitalizations, all-cause mortality, or primary care utilization in the high-risk cohort, it did result in an 11.7 percentage point increase in the primary endpoint of influenza vaccination rates by January 2024 compared with the usual care group. The key finding from this secondary analysis was that messaging, particularly repeated letters and cardiovascular gain framing, may have been more effective in participants without diabetes than in those with diabetes. This pattern was consistent across all message types and mirrored the results of the NUDGE-FLU trial that targeted individuals aged 65 years and older.^9^

The subanalysis of the NUDGE-FLU-CHRONIC trial provided three major insights. First, the effectiveness of text-based nudges. Compared with NUDGE-FLU, electronic nudge letters were consistently effective in increasing influenza vaccination rates among young and middle-aged adults with chronic diseases, regardless of the message type. However, among patients with diabetes, who tended to have higher baseline vaccination rates, the effect was somewhat weaker. This suggests that text-based nudges have significant potential to improve vaccination uptake, especially in populations with baseline low vaccination rates that are “nudge-able.”

Second, the effectiveness of specific message types, as observed in NUDGE-FLU, repeated letters and cardiovascular gain framing were particularly effective. Repeated letters demonstrated consistent results across studies, indicating that a simple reminder function—sending a standard letter twice—can be highly effective. A unique aspect of this study was the effectiveness of cardiovascular gain framing in communicating the secondary benefits of vaccination in preventing cardiovascular diseases, especially in young and middle-aged cohorts.

Third, the influence of patient factors on the effectiveness of messages is closely related to message type. While the subanalysis of cardiovascular diseases in the NUDGE-FLU-CHRONIC trial is yet to be completed, the NUDGE-FLU trial’s subanalysis showed that participants with cardiovascular diseases responded more strongly to messaging than those without.^10^ This suggests that certain messages may be more effective depending on the presence of underlying conditions. It also indicates the potential for developing more tailored text-based nudge messages that address individual patient characteristics beyond the underlying conditions.

Although the authors presented excellent results, it is important to consider certain factors when interpreting the findings. In particular, in the context of influenza vaccination research, specific health care policies and contextual factors of different countries, such as Denmark, should be taken into account, including the message content, target population, and specific circumstances there. For example, influenza vaccination is provided for free and digital messages are sent without requiring additional consent. NUDGE-FLU uses an opt-out system in which messages are sent by default. In contrast, the Walmart Megastudy conducted by Milkman et al^6^ required participants to opt for message receipt in the previous year or earlier. Ethical differences such as the level of consent required in randomized controlled trials have become an important consideration when implementing such studies in other countries. Additionally, while the repeated message in this study was sent 10 days after the initial message, further clarification is needed regarding the timing differences between the research groups. These differences could affect future implementation, depending on the regional context. The content of messages should also be carefully tailored to specific contexts. For instance, the cardiovascular gain framing message in this study stated, “In addition to its protection against influenza infection, influenza vaccination also seems to protect against cardiovascular diseases such as heart attacks and heart failure.” It is important to recognize that different regions may interpret this message differently, particularly in areas where stroke is more prevalent or infectious diseases take priority over cardiovascular concerns. As seen in the NUDGE-FLU2 study,^11^^,^^12^ which followed the NUDGE-FLU trial and targeted individuals aged 65 years and older, the effects of framing were not as pronounced, suggesting that we should be cautious about overestimating the impact of each framing technique.

Despite its limitations, this approach co2uld be applied not only to other vaccines like respiratory syncytial virus but also to nonrespiratory vaccines like the shingle vaccine. Furthermore, the insights from this research may be applied to long-term therapies such as incliciran, a small interfering ribonucleic acid therapy for lipid management, which likely face adherence challenges similar to those encountered with vaccines. The differing motivations between infectious and noninfectious diseases will continue to provide valuable insights. In conclusion, the authors meticulously analyzed and discussed their findings, contributing significant data from a behavioral economics perspective. Education on behavioral economics and decision-making should be integrated into the training of all clinicians.^13^ These outstanding studies and their results are likely to be critically examined in the future.

## Funding support and author disclosures

This work was supported by Japan Agency for Medical Research and Development (AMED) under Grant Number JP23rea522009 (to Dr Mizuno). The authors have reported that they have no relationships relevant to the contents of this paper to disclose.
